# Analysis of early mesothelial cell responses to *Staphylococcus epidermidis* isolated from patients with peritoneal dialysis-associated peritonitis

**DOI:** 10.1371/journal.pone.0178151

**Published:** 2017-05-24

**Authors:** Amanda L. McGuire, Kieran T. Mulroney, Christine F. Carson, Ramesh Ram, Grant Morahan, Aron Chakera

**Affiliations:** 1 Translational Renal Research Group, Harry Perkins Institute of Medical Research, Nedlands, Western Australia, Australia; 2 School of Medicine and Pharmacology, University of Western Australia, Crawley, Western Australia, Australia; 3 Centre for Diabetes Research, Harry Perkins Institute of Medical Research, Nedlands, Western Australia, Australia; 4 Department of Renal Medicine, Sir Charles Gairdner Hospital, Nedlands, Western Australia, Australia; Hospital Universitario de la Princesa, SPAIN

## Abstract

The major complication of peritoneal dialysis (PD) is the development of peritonitis, an infection within the abdominal cavity, primarily caused by bacteria. PD peritonitis is associated with significant morbidity, mortality and health care costs. *Staphylococcus epidermidis* is the most frequently isolated cause of PD-associated peritonitis. Mesothelial cells are integral to the host response to peritonitis, and subsequent clinical outcomes, yet the effects of infection on mesothelial cells are not well characterised. We systematically investigated the early mesothelial cell response to clinical and reference isolates of *S*. *epidermidis* using primary mesothelial cells and the mesothelial cell line Met-5A. Using an unbiased whole genome microarray, followed by a targeted panel of genes known to be involved in the human antibacterial response, we identified 38 differentially regulated genes (adj. *p*-value < 0.05) representing 35 canonical pathways after 1 hour exposure to *S*. *epidermidis*. The top 3 canonical pathways were TNFR2 signaling, IL-17A signaling, and TNFR1 signaling (adj. *p*-values of 0.0012, 0.0012 and 0.0019, respectively). Subsequent qPCR validation confirmed significant differences in gene expression in a number of genes not previously described in mesothelial cell responses to infection, with heterogeneity observed between clinical isolates of *S*. *epidermidis*, and between Met-5A and primary mesothelial cells. Heterogeneity between different *S*. *epidermidis* isolates suggests that specific virulence factors may play critical roles in influencing outcomes from peritonitis. This study provides new insights into early mesothelial cell responses to infection with *S*. *epidermidis*, and confirms the importance of validating findings in primary mesothelial cells.

## Introduction

The prevalence of end stage kidney disease (ESKD) is increasing due to an aging population and a rise in the incidence of diabetes and hypertension [1–3]. It has been estimated that 1.9 million people worldwide are undergoing renal replacement therapy [1], which is associated with significant healthcare costs [4]. Peritoneal Dialysis (PD) is a commonly used treatment modality for ESKD that requires a permanent catheter placed into the abdomen. The most frequent complication of PD is the development of peritonitis [5, 6], an infection within the abdomen, which is responsible for the majority of treatment failures and significant mortality [7]. Gram positive microorganisms account for 60–70% of PD peritonitis cases, with coagulase-negative staphylococci (CoNS) the predominant pathogens [8, 9]. *S*. *epidermidis* account for approximately 50-70% of CoNS causing PD peritonitis [10–12].

The initial phase of the host response to peritonitis is mediated by mesothelial cells–a specialised single cell layer that covers the visceral and parietal surfaces of organs within the abdominal and chest cavities [13]. Mesothelial cells are highly metabolically active, recognize pathogen-associated molecular pathways, and can produce numerous cytokines [14, 15]. Despite the importance of these cells, few studies have assessed how mesothelial cells respond to pathogens causing peritonitis and most have been limited to analysis of individual signalling molecules or genes of interest.

**Fig 1:**
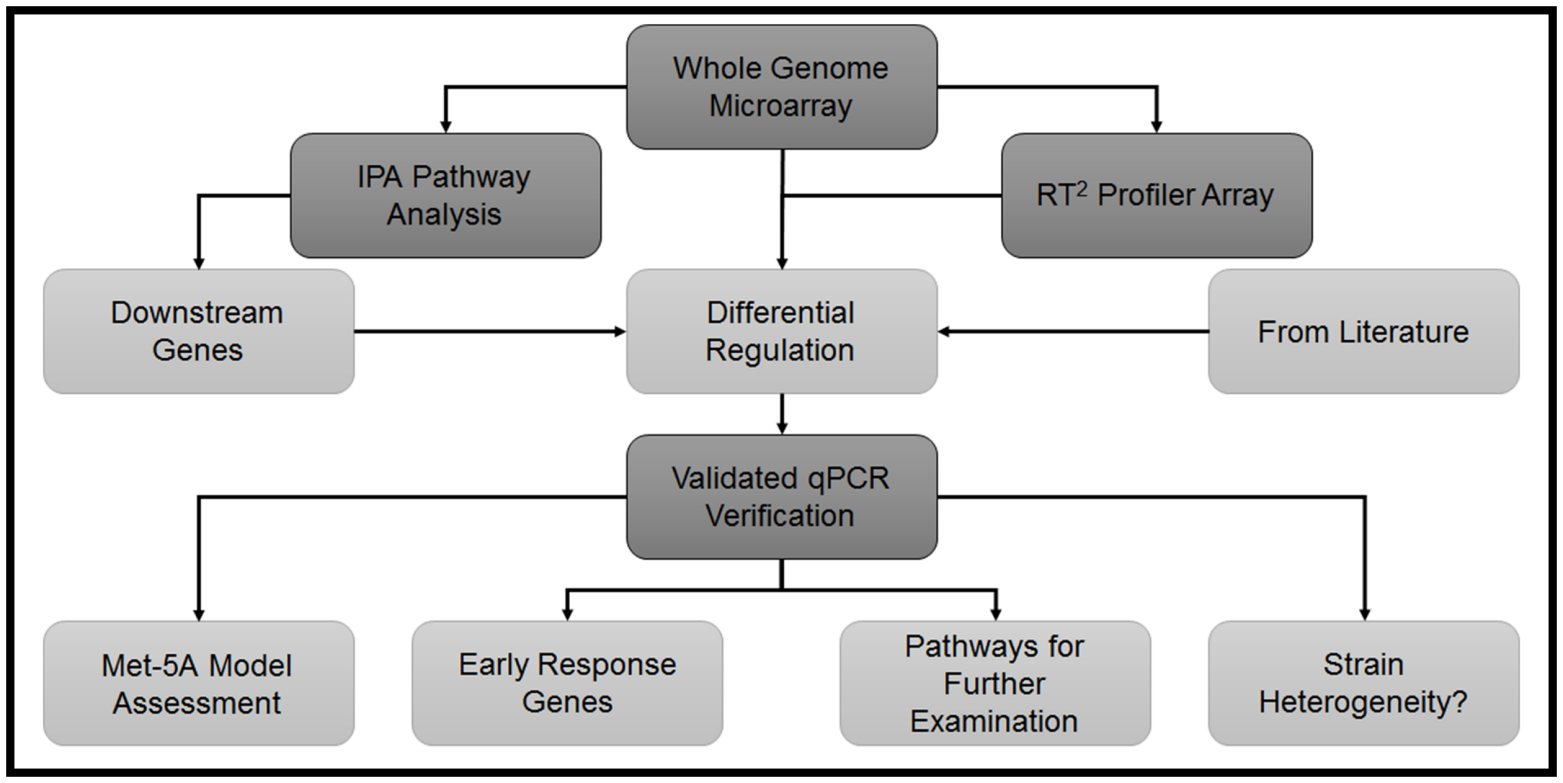
Flow chart demonstrating the experimental approach. Experimental steps are shown in dark grey and analysis and experimental questions are shown in light grey. IPA = Ingenuity Pathway Analysis.

In this study, we demonstrate that *S*. *epidermidis* induces a complex series of changes in gene transcription in mesothelial cells within 1 hour of bacterial exposure. An overview of the experimental approach is shown in Fig 1. These changes affect pathways associated with tumor necrosis factor (TNF) and Toll-like receptor (TLR) signaling. Mesothelial cell responses to *S*. *epidermidis* infection vary between isolates and between primary cells and the Met-5A mesothelial cell line for a number of key genes, including TNF. These findings provide new insights into the early host response to PD peritonitis and highlight the importance of validating data from mesothelial cell lines in primary mesothelial cells.

## Materials and methods

### Bacterial strains

*S*. *epidermidis* reference isolates ATCC^®^ 14990 and ATCC^®^ 12228 (American Type Culture Collection (ATCC), Manassas, VA, USA), and clinical *S*. *epidermidis* isolates cultured from PD effluent (C015 to C019) were provided by PathWest Laboratory Medicine, Western Australia. Identities were confirmed by MALDI-TOF using a MALDI Biotyper Reference Library (Bruker Daltonics, Bremen, Germany) prior to use. Bacteria were grown on 5% sheep blood agar (BA) plates at 37°C/5% CO_2_, and a single colony chosen for expansion overnight in Luria-Bertani broth (LB; LB-Miller, BD Difco^™^, Cat. No. 244620) at 37°C at 200 rpm. Standardised bacterial suspensions were prepared to a density 1.0–1.5 x 10^8^ colony forming units (cfu)/mL using the approximation 0.1 OD_600_ = 1 x 10^8^ cfu/mL using a spectrophotometer (NanoPhotometer^™^, Implen, Munich, Germany), or to 0.5 McFarland Standard (~1.5 x 10^8^ cfu/mL) using a Sensititre^™^ Nephelometer (Thermo Fisher Scientific). Viable counts were determined by serial dilution in phosphate buffered saline (PBS) and plating on BA plates.

### Cell culture conditions

Human primary mesothelial cells, derived from adult omental tissue and pooled from multiple donors, were obtained from Zen-Bio Inc. (Research Triangle Park, NC, USA; Cat. No. DMES-F-SL). During resuscitation from liquid nitrogen, primary mesothelial cells were cultured in Mesothelial Cell Growth Medium (Zen-Bio Inc.; Cat. No. MSO-1), consisting of Medium 199, fetal bovine serum (FBS), human epidermal growth factor, penicillin, streptomycin, and amphotericin B (proprietary formula). All gene expression experiments were conducted in Dulbecco’s Modified Eagle’s Medium (DMEM) containing 4500 mg/L glucose (Sigma-Aldrich, St. Louis, MO USA) and supplemented with 4 mM L-glutamine (Sigma-Aldrich), 200U/mL penicillin/0.2 mg/mL streptomycin (Sigma-Aldrich), 15% FBS (Bovogen Biologicals Pty Ltd, Keilor East, Victoria, Australia; Cat. No. SFBS-F) and 0.4 μg/mL hydrocortisone (Sigma-Aldrich) [16]. Met-5A mesothelial cells (ATCC^®^ CRL-9444) were cultured in the same formulation of DMEM as the primary mesothelial cells, but without hydrocortisone and using 10% FBS [17].

### Bacterial challenge conditions

Confluent cells were serum starved in the absence of antibiotics for 18 hours prior to incubation with bacteria. Standardised bacterial suspensions (~1 x 10^8^ cfu/mL) were diluted ^1^/_10_ in the appropriate antibiotic-free cell culture media to give ~1 x 10^7^ cfu/mL, of which 2 mL was co-incubated with cells for 1 hour at 37°C/5% CO_2_. For dose-response experiments, bacterial suspensions were standardized to ~1 x 10^9^ cfu/mL, serially diluted in LB broth then diluted ^1^/_10_ in cell-culture media, as described above. Met-5A cells were also exposed to lipoteichoic acid (LTA) from *Staphylococcus aureus* (Sigma; Cat. No. L2515), the primary component of the Gram positive cell wall, at 10 μg/mL in antibiotic-free DMEM. All test conditions were set up in triplicate in 6 well plates (Falcon^®^ by Corning, Corning NY USA). Control wells contained mesothelial cells with media alone, or media containing 10% LB.

### RNA isolation from primary mesothelial cells and the Met-5A cell line

Following co-incubation with bacteria, mesothelial cell monolayers were washed with PBS pre-warmed to 37°C. Mesothelial cells for RT^2^ PCR array and qPCR experiments were treated with RNAprotect Cell Reagent (Qiagen GmbH, Hilden, Germany), with 300 μL PBS and 1.5 mL RNAprotect added per well. RNA was isolated using the RNeasy Plus Mini kit (Qiagen) with gDNA Eliminator spin columns. RNA was quantified using the Caliper LabChip GXII (Perkin Elmer, Waltham, MA, USA) at the Australian Genome Research Facility (AGRF), Perth, Australia or a NanoDrop 2000 (Thermo Fisher Scientific Inc., Wilmington, DE, USA). RNA quality was determined by assessment of A_260_/A_280_ and A_260_/A_230_ ratios.

### Viability of mesothelial cells by flow cytometry

Following co-incubation with bacteria, cell monolayers were washed with warm PBS and harvested using a 0.05% trypsin-EDTA solution (Sigma-Aldrich). Cells were stained with LIVE/DEAD^®^ Fixable Near-IR Dead Cell Stain Kit (Thermo Fisher Scientific) as per the manufacturer’s protocols, fixed in 4% paraformaldehyde, and acquired in technical triplicate using a FACSCanto^™^ II flow cytometer (BD Biosciences, Franklin Lakes, NJ, USA). Data were exported in FCS version 3.1, and analysis was completed using FlowJo Version 10.0.08 (FlowJo LLC., Ashland OR USA) and Prism Version 6.0b (GraphPad Software, San Diego CA USA). Comparisons between unexposed and bacteria-exposed samples were made using unpaired t-tests.

### Illumina HT-12 v4 human genome microarray

RNA samples (biological triplicates) from *S*. *epidermidis*-infected primary mesothelial cells (and controls) that met quality control requirements were sent to the AGRF, Melbourne, Australia for microarray processing using the HT-12 v4 human genome microarray (Illumina, Inc., San Diego, CA USA). A total hybridisation volume of 15 μL was prepared for each sample, and loaded per microarray on the Ilumina HumanHT-12 Expression BeadChip. Hybridisation was at 58°C for 16 hours on a rocking platform. Following hybridisation, samples were washed as per manufacturer’s instructions, coupled with Cy3, and scanned in the Illumina iScan Reader, with output produced by GenomeStudio version 1.9.0. Using R (version 3.1.2) [18]. The data underwent quality control through the Bioconductor [19] packages *arrayQualityMetrics* [20], *made4* [21], *lumi* [22] and *limma* [23]. The detectable probe ratio of each probe was calculated, and all probes with a detection *p*-value of less than 0.01 were removed, and relative quality weights were estimated for each microarray. A linear model was fitted contrasting the control samples relative to the *S*. *epidermidis* samples, resulting in differentially expressed genes under a false discovery rate of 5%. Significantly differentially regulated genes had a Benjamini-Hochberg adjusted *p*-value < 0.05 [24]. Microarray data files for *S*. *epidermidis* ATCC^®^ 14990 *and S*. *epidermidis* ATCC^®^ 12228 have been deposited at https://researchdataonline.research.uwa.edu.au/handle/123456789/3381.

### Human antibacterial response RT^2^ PCR array

0.5 μg RNA from primary mesothelial cells and Met-5A cells exposed to 10^7^ cfu/mL bacteria, LTA (10 μg/mL) or controls for 1 hour was processed for the Qiagen ‘Human Antibacterial Response’ RT^2^ PCR array (Cat. No. PAHS-148Z) according to the manufacturer’s instructions. Real-time PCR cycling was performed using a StepOnePlus^™^ Real-Time PCR System (Thermo Fisher Scientific). Data were normalised to the reference gene, RPLP0. Data were presented as fold change, with >2-fold considered up-regulation and <-2-fold considered down-regulation. RT^2^ PCR array data have been deposited at https://researchdataonline.research.uwa.edu.au/handle/123456789/3381.

### qPCR validation of gene expression

cDNA was synthesized from 1 μg total RNA using the iScript^™^ cDNA Synthesis kit (Bio-Rad; Cat. No. 1708891) in a 20 μL reaction volume, according to the manufacturer’s instructions. Gene expression findings of key genes identified by microarray and RT^2^ PCR arrays were validated by qPCR using a StepOnePlus^™^ real-time PCR system (Thermo Fisher Scientific), wet-lab validated PrimePCR^™^ Gene Expression Probe assays (Bio-Rad) and were conducted following MIQE guidelines [25]. Genes assayed by qPCR were TNF (unique assay ID dHsaCPE5190842; Bio-Rad), TLR4 (dHsaCPE5030581), CCL5 (dHsaCPE5050154), ZFP36 (dHsaCPE5191899), EDN1 (dHsaCPE5053386) and ITLN1 (dHsaCPE5041777). The reference gene was RPLP0 (dHsaCPE5031575). Samples were assayed in a minimum of biological triplicates, assayed in technical triplicate, and data were analysed using the comparative Ct method (ΔΔCt), with results reported as the fold-change in gene expression (2^-ΔΔCt^) relative to the DMEM/10% LB control.

### Analysis of differentially expressed genes using Ingenuity Pathway Analysis

Genes identified by microarray analysis as significantly differentially expressed (fold change > ±1.5, adj. *p*-value < 0.05) were subjected to Qiagen’s Ingenuity^®^ Pathway Analysis (IPA^®^, Qiagen Redwood City, www.qiagen.com/ingenuity, IPA v1.07) to determine canonical pathways, upstream regulators and networks significantly enriched for these genes. Ten of the 38 differentially expressed genes were excluded from IPA (5 duplicate genes, 4 uncharacterized genes, and 1 gene below the IPA default criteria for pathway analysis). The remaining 28 genes were mapped using the Hugo Gene Nomenclature Committee (HGNC) database and expression differences were uploaded to IPA as fold changes. The Core Analysis function was performed and a right-tailed Fisher’s exact test was used to calculate the significance of each pathway or biological function. A Benjamini-Hochberg adjusted *p* < 0.01 was treated as significant [24].

## Results

### Mesothelial cell viability following bacterial infection

**Fig 2:**
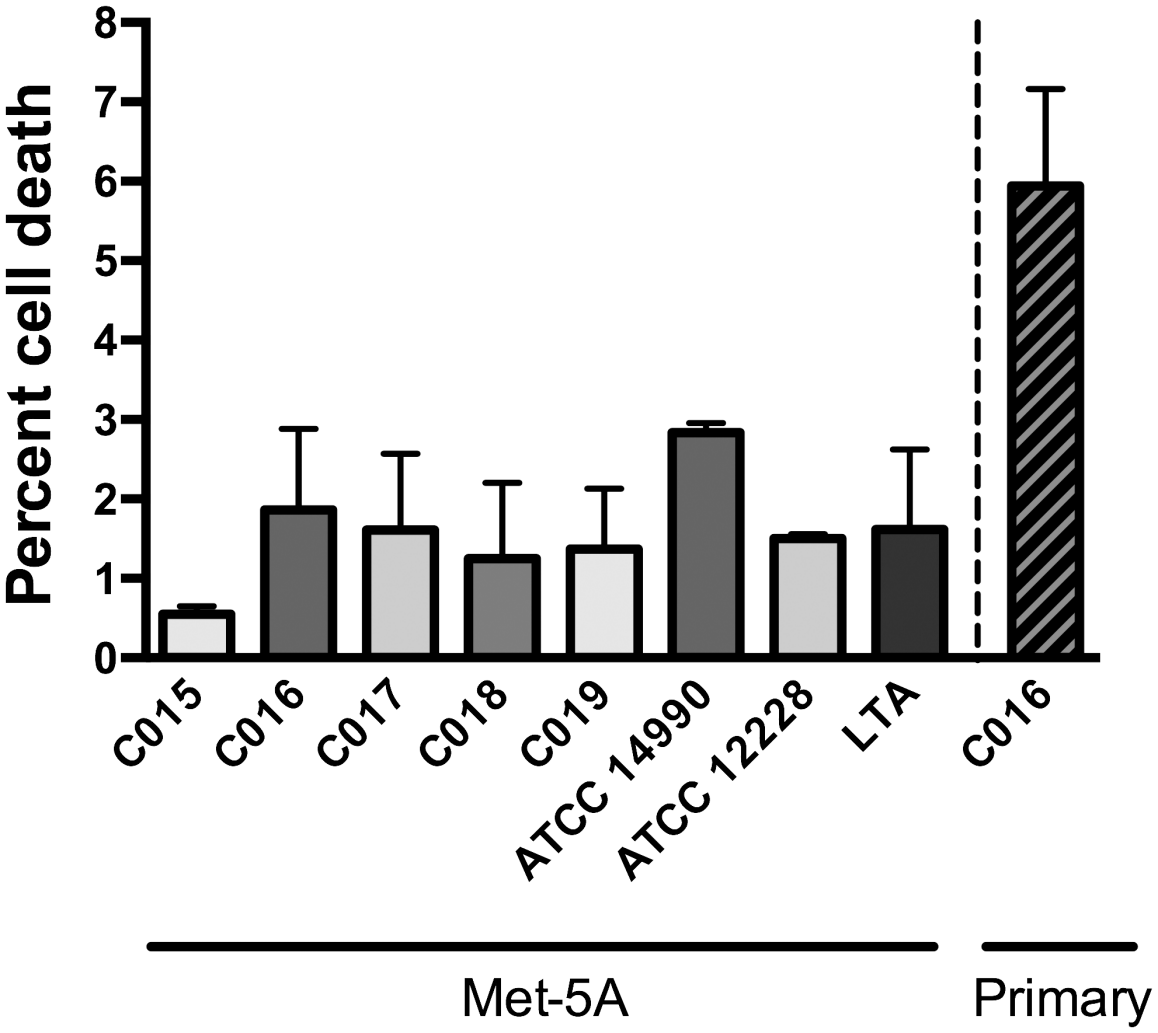
Viability of Met-5A and primary mesothelial cells exposed to *S*. *epidermidis* and lipoteichoic acid. Confluent Met-5A mesothelial cells were exposed to 10^7^ cfu/mL of two *S*. *epidermidis* reference isolates (ATCC^®^ 14990 and ATCC^®^ 12228), five *S*. *epidermidis* clinical isolates from PD peritonitis patients (C015, C016, C017, C018, C019) and 10 μg/mL lipoteichoic acid (LTA) for 1 hour at 37°C. Confluent primary mesothelial cells were exposed to 10^7^ cfu/mL of the clinical *S*. *epidermidis* isolate C016. Viability was determined using flow cytometry and a LIVE/DEAD^®^ Fixable Near-IR Dead Cell Stain, and data reported as the mean percentage of cell death across a minimum of biological triplicates (error bars are standard deviation). There was no statistically significant (*p* < 0.05) difference in the percent of cell death between any of the samples and the media control.

*In vitro* cultured mesothelial monolayers were exposed to 10^7^ cfu/mL of five clinical isolates of *S*. *epidermidis*, two reference isolates of *S*. *epidermidis* and LTA (10 μg/mL) for 1 hour. Following exposure, 0.5–7.5% of cells assayed were non-viable (Fig 2), with no significant (*p* < 0.05) difference in mesothelial cell viability between any of the samples and the media control. Therefore, a 1 hour exposure with 10^7^ cfu/mL *S*. *epidermidis* was selected for gene expression studies to maximise the strength of the bacterial challenge signal whilst avoiding enrichment of apoptotic/necrotic gene expression pathways, and limiting variation induced by growth of the bacterial inoculum.

### Primary mesothelial cell human genome microarray

**Fig 3:**
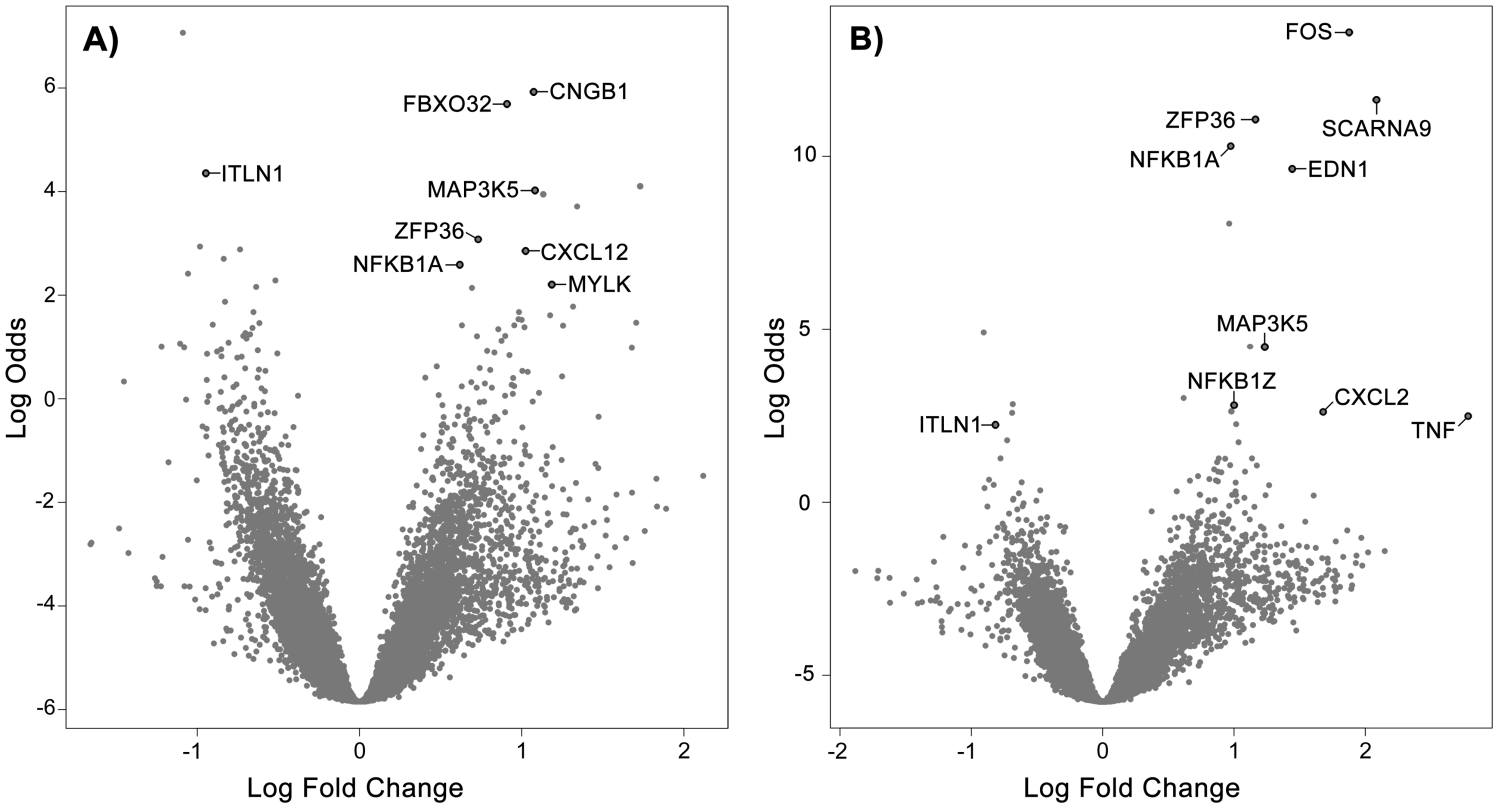
Volcano plots showing differentially expressed genes following incubation of primary mesothelial cells with *S*. *epidermidis* isolates for 1 hour. Volcano plots showing differentially regulated genes (adj. *p*-value < 0.05) following exposure of primary mesothelial cells to *S*. *epidermidis* ATCC^®^ 14990 (A) or *S*. *epidermidis* ATCC^®^ 12228 (B). A positive Log Fold Change indicates up-regulation; a negative Log Fold Change indicates down-regulation. The Log Odds (B value) is the log of the probability that a gene is differentially expressed. A Log Odds value of 0 corresponds to a 50–50 chance that the gene is differentially expressed.

Nineteen significantly differentially regulated genes (adj. *p*-value < 0.05) were identified in each *S*. *epidermidis* isolate (for a total of 38 genes of interest), with 25 genes up-regulated and 13 genes down-regulated (Tables 1 and 2, respectively). Four genes were significantly differentially regulated in response to both *S*. *epidermidis* isolates: MAP3K5, NFKBIA and ZFP36 (up-regulated), and ITLN1 (down-regulated) (Fig 3, and Tables 1 and 2).

**Table 1:** Primary mesothelial cell genes significantly up-regulated by *S*. *epidermidis* at 1 hour.

Isolate^1^	Target ID	Adjusted *p*-value^2^	B value (Log-Odds)^3^	log_2_FC^4^
Se2	FOS	5.51E-07	13.571796	1.879734
Se2	SCARNA9	2.89E-06	11.648745	2.086159
Se2	**ZFP36**^5^	4.77E-06	11.078705	1.162754
Se2	**NFKBIA**	9.36E-06	10.306066	0.978205
Se2	EDN1	1.70E-05	9.644657	1.441711
Se2	EGR1	9.82E-05	8.060662	0.963757
Se1	CNGB1^6^	4.17E-03	5.918297	1.071359
Se1	FBXO32	4.17E-03	5.705710	0.909449
Se2	CYP4B1	4.78E-03	4.493523	1.235685
Se2	**MAP3K5**	4.78E-03	4.511479	1.120886
Se1	CNGB1^6^	1.36E-02	4.105234	1.730148
Se1	**MAP3K5**	1.36E-02	4.026606	1.076436
Se1	VNN3	1.36E-02	3.953305	1.132151
Se1	LOC644422	1.57E-02	3.713648	1.341445
Se2	IER3	2.46E-02	3.026337	0.614906
Se2	NFKBIZ	2.68E-02	2.809053	0.998651
Se2	CXCL2	2.74E-02	2.613680	1.670304
Se2	LOC338758	2.74E-02	2.642575	0.978880
Se1	CXCL12	2.82E-02	2.856812	1.023841
Se1	**ZFP36**	2.82E-02	3.086259	0.733774
Se2	TNF	2.87E-02	2.518083	2.779602
Se2	LOH3CR2A	3.25E-02	2.261506	1.014301
Se1	**NFKBIA**	3.26E-02	2.594352	0.618322
Se1	KLF2	4.06E-02	2.140259	0.693826
Se1	MYLK	4.06E-02	2.208976	1.189960

^1^ Se1: *S*. *epidermidis* ATCC^®^ 14990. Se2: *S*. *epidermidis* ATCC^®^ 12228.

^2^ Benjamini-Hochberg adjusted *p*-value < 0.05 is considered statistically significant.

^3^ The B value is the Log-Odds that the gene is differentially expressed.

^4^ log_2_FC = log_2_ Fold Change

^5^ Genes shown in bold font were significantly up-regulated by both *S*. *epidermidis* isolates.

^6^ CNGB1 was represented on the microarray twice, with both probes significantly up-regulated by Se1.

**Table 2:** Primary mesothelial cell genes significantly down-regulated by *S*. *epidermidis* at 1 hour.

Isolate^1^	Target ID	Adjusted *p*-value^2^	B value (Log-Odds)^3^	log_2_FC^4^
Se1	KIAA1644	2.57E-03	7.065969	-1.088678
Se2	ANG	3.74E-03	4.908400	-0.905677
Se1	**ITLN1**^5^	1.36E-02	4.356518	-0.947596
Se2	SERTAD1	2.68E-02	2.851482	-0.685160
Se2	LOC645638	2.74E-02	2.592000	-0.690900
Se1	HAS1	2.82E-02	2.887356	-0.735746
Se1	PAQR9	2.82E-02	2.941605	-0.983196
Se1	ERF	3.09E-02	2.706018	-0.836912
Se2	BAIAP2	3.25E-02	2.251291	-0.811500
Se2	**ITLN1**^5^	3.25E-02	2.247736	-0.823852
Se1	LOC100129975	3.74E-02	2.417200	-1.057123
Se1	LMCD1	4.06E-02	2.161595	-0.636474
Se1	SLC20A1	4.06E-02	2.288993	-0.517624

^1^ Se1: *S*. *epidermidis* ATCC^®^ 14990. Se2: *S*. *epidermidis* ATCC^®^ 12228.

^2^ Benjamini-Hochberg adjusted *p*-value < 0.05 is considered statistically significant.

^3^ The B value is the Log-Odds that the gene is differentially expressed.

^4^ log_2_FC = log_2_ Fold Change

^5^ ITLN1 was significantly down-regulated by both *S*. *epidermidis* isolates.

### Pathway analysis of differentially expressed genes

**Fig 4:**
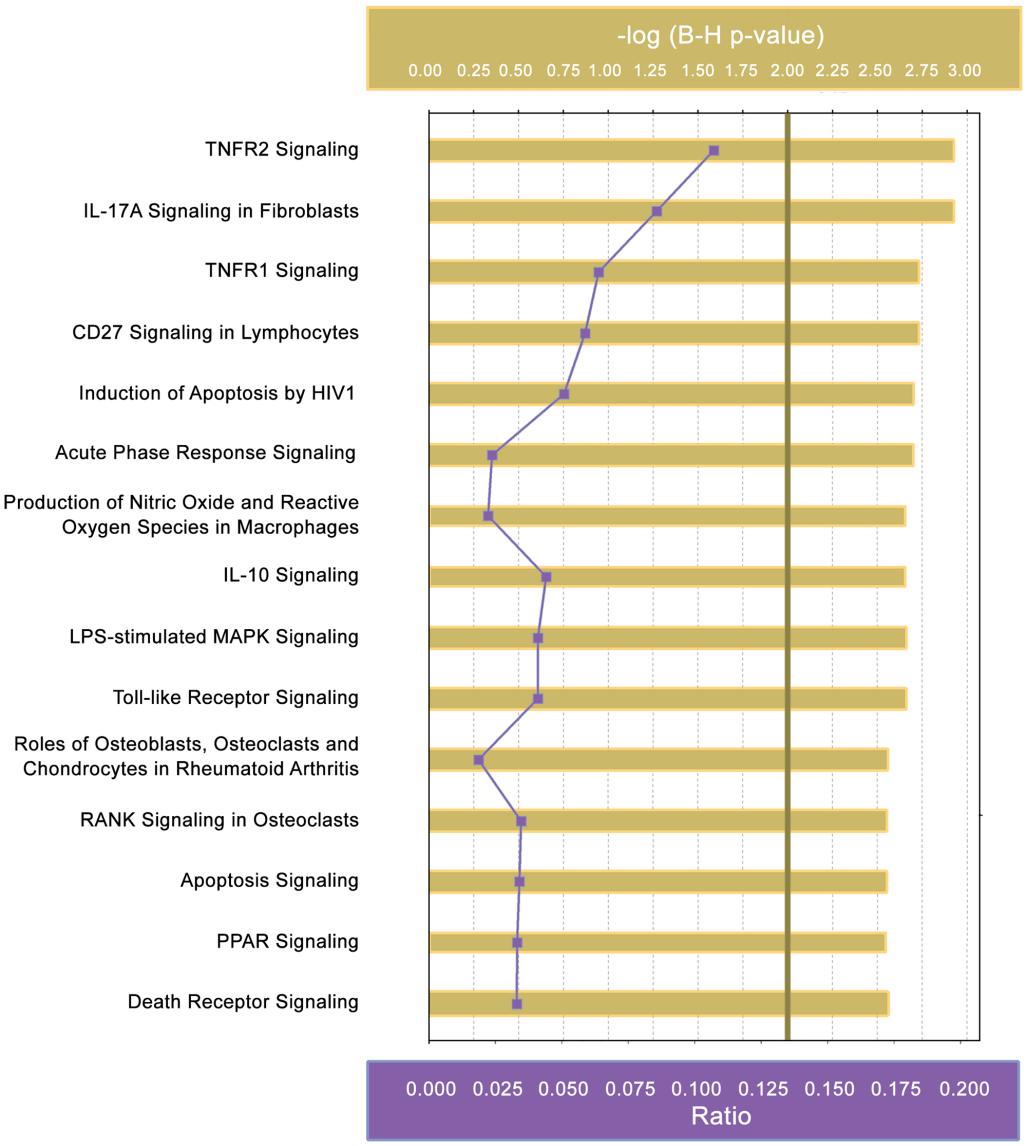
Canonical pathways represented by the differentially expressed genes following incubation of primary mesothelial cells with *S*. *epidermidis* for 1 hour. 28 differentially expressed genes identified by microarray following incubation of *S*. *epidermidis* with primary mesothelial cells were analysed using Ingenuity Pathway Analysis (IPA) and 35 canonical pathways were represented in our dataset. The top 15 canonical pathways are shown above, with the full list of canonical pathways shown in S1 Table. A -log(B-H *p*-value) (shown in gold) of >2 represents data with an adjusted *p*-value < 0.01 (threshold for significance shown as a vertical line at 2.00). The ratio (shown in purple) indicates the proportion of differentially expressed genes relative to the total number of genes in each pathway.

**Fig 5:**
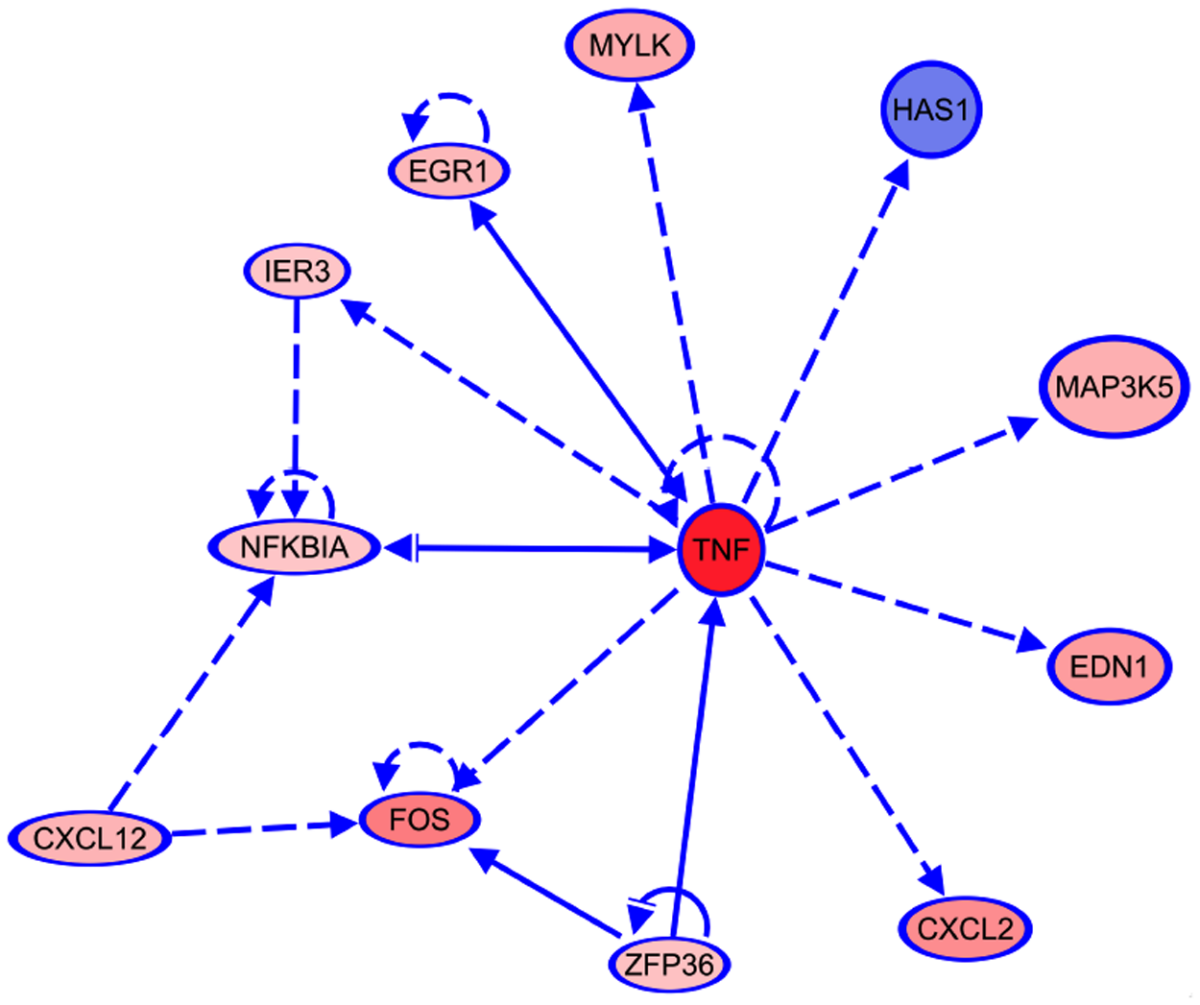
TNF is an upstream regulator of ten of the differentially regulated genes. The connections between nodes represent direct (solid lines) and indirect (dashed lines) relationships between genes, as supported by information in the IPA database. Up-regulated genes are shaded red, and down-regulated genes are shaded blue, with the intensity of the colour indicative of the magnitude of regulation. Feedback loops indicate auto regulation.

To ascertain the relationships between genes identified by microarray, and aid in selection of further genes for study, twenty-eight genes that were significantly differentially expressed in primary mesothelial cells following *S*. *epidermidis* exposure were analysed using Qiagen’s Ingenuity Pathway Analysis (IPA) software. Ten of the 38 differentially expressed genes were excluded from IPA analysis, as described in the Materials and Methods. Thirty-five canonical pathways were significantly represented (adj. *p*-value < 0.01) in our dataset (S1 Table), with the top 15 canonical pathways shown in Fig 4. The three most significant pathways were TNFR2 signaling, IL-17A Signaling in Fibroblasts, and TNFR1 signaling, with other notable pathways including TLR signaling, and apoptosis signaling. TNF was both a differentially expressed gene, and an upstream regulator of 10 of the differentially expressed genes (CXCL2, EDN1, EGR1, FOS, HAS1, IER3, MAP3K5, MYLK, NFKBIA, ZFP36) as determined by microarray (Fig 5).

### RT^2^ human antibacterial response PCR array

**Fig 6:**
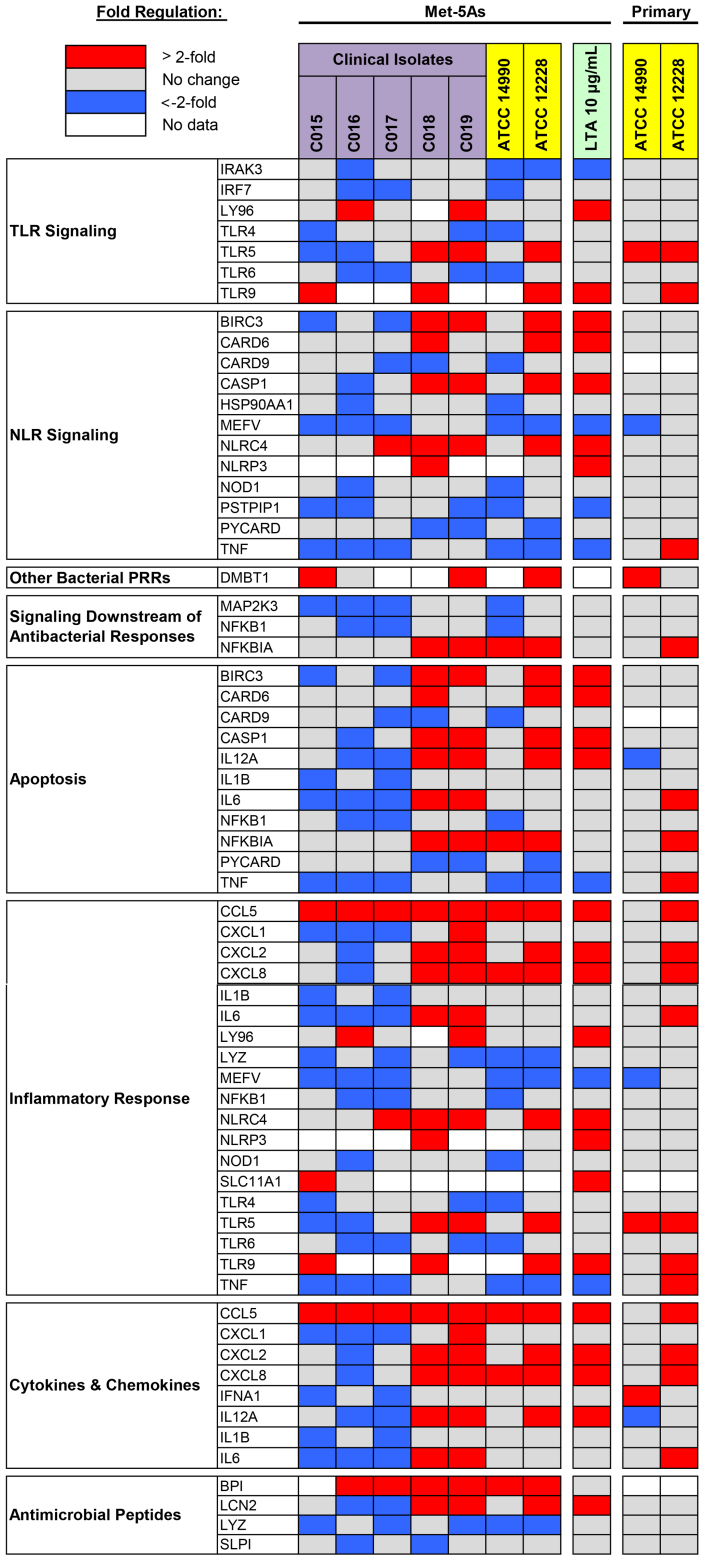
Changes in mesothelial cell gene expression in response to *S*. *epidermidis*. Confluent Met-5A or primary mesothelial cells were exposed to 10^7^ cfu/mL isolates of *S*. *epidermidis* or 10 μg/mL lipoteichoic acid (LTA) for 1 hour. Changes in gene expression were analysed using the RT^2^ human antibacterial response PCR array. 36 of the 84 genes on the RT^2^ panel were differentially regulated (>2-fold) in ≥2 *S*. *epidermidis* isolates and are shown grouped by category.

Following analysis of the microarray data, the Qiagen RT^2^ human antibacterial response PCR array was used to further assess changes in gene expression. Each RT^2^ PCR array contained 84 genes and across the conditions tested in Met-5A cells, 478 of the 588 genes were expressed in the 7 *S*. *epidermidis* arrays. There were 36 genes (7.5% of the 478 genes) up-regulated (3 to 26 fold), and 32 genes (6.7%) down-regulated (3 to 33 fold). 36 genes were differentially regulated (>2-fold) in two or more *S*. *epidermidis* isolates (Fig 6). Twelve genes were differentially regulated in a single isolate, 27 genes did not show a change in expression, and no expression data was available for 9 genes, suggesting these genes are not expressed by mesothelial cells under the conditions tested.

Primary mesothelial cells showed lower magnitude changes in gene expression 1 hour after infection with *S*. *epidemidis* relative to Met-5A cells. There were 145 genes expressed across two reference isolates, with 4 genes (2.8%) up-regulated 3 to 19 fold, and 2 genes (1.3%) down-regulated 3 to 11 fold. TNF was the most highly up-regulated gene in primary mesothelial cells. Results for TNF expression in Met-5As differed significantly from primary mesothelial cells, with TNF consistently down-regulated in Met-5A cells (Fig 6).

### qPCR validation

The selection of genes for qPCR validation was determined by a multi-factorial approach, as outlined in Fig 1. Criteria for inclusion were: magnitude of expression fold change across microarray and RT^2^ PCR array experiments, consistency of differential expression across conditions, differences between Met-5A and primary mesothelial cell responses, biological plausibility (from existing literature), and potential consequence in the context of PD peritonitis. Based on these criteria, 6 genes were selected for further investigation: CCL5 (RANTES), TLR4, TNF, ZFP36, EDN1 and ITLN1. Dose response experiments in primary and Met-5A mesothelial cells were conducted using the clinical *S*. *epidermidis* isolate C016 at 10^8^ cfu/mL, 10^6^ cfu/mL and 10^4^ cfu/mL for 1 hour (Table 3).

**Table 3:** Changes in mesothelial cell gene expression of CCL5, TLR4, TNF, ZFP36, EDN1 and ITLN1 1 hour after infection with *S*. *epidermidis*.

Method	*S*. *epidermidis* Isolate	cfu/mL	Fold change in gene expression^1^								
			Primary mesothelial cells						Met-5A mesothelial cells		
			CCL5	TLR4	TNF	ZFP36^2^	EDN1^2^	ITLN1^2^	CCL5	TLR4	TNF
Microarray	ATCC 14990	10^7^	1.30	ND^3^	-1.18	1.66	1.52	-1.93	−^4^	−^4^	−^4^
	ATCC 12228	10^7^	1.32	ND^3^	6.87	2.24	2.72	-1.77	−^4^	−^4^	−^4^
RT^2^ Array	C016	10^7^	−^4^	−^4^	−^4^	−^4^	−^4^	−^4^	6.73	-1.81	-6.16
	ATCC 14990	10^7^	1.68	-1.08	-1.23	−^4^	−^4^	−^4^	2.64	-2.67	-5.74
	ATCC 12228	10^7^	2.25	-1.57	19.43	−^4^	−^4^	−^4^	6.41	-1.63	-3.00
qPCR	C016	10^8^	1.11	-1.04	14.52	1.48	1.47	-1.41	1.54	2.28	-1.59
	C016	10^6^	1.13	-1.35	1.07	-1.02	-1.13	-1.25	1.79	2.31	-1.30
	C016	10^4^	1.18	-1.18	-1.10	-1.25	-1.18	-1.25	1.70	2.40	-1.01

^1^ Fold change relative to the media control.

^2^ −, Gene not present on the RT^2^ PCR array.

^3^ ND, Not detected.

^4^ −, Not tested.

Gene expression in primary mesothelial cells exposed to high doses of *S*. *epidermidis* was consistent between microarray studies, RT^2^ PCR arrays and qPCR for CCL5, ZFP36 and EDN1 (up-regulated) and TLR4 and ITLN1 (down-regulated). Expression of TNF showed a dose-dependent response in primary mesothelial cells, with 14-fold to 19-fold increases in TNF expression with high doses of *S*. *epidermidis*. Expression of CCL5 by Met-5A mesothelial cells was in agreement with primary cells. However aberrant expression of TLR4 (inconsistent results) and TNF (opposing results) in Met-5A mesothelial cells was noted.

## Discussion

Peritonitis caused by coagulase-negative staphylococci is a common complication of peritoneal dialysis therapy and is associated with significant morbidity and mortality [8, 26]. Mesothelial cells are a first line of defense in the peritoneal cavity and the response of these cells to the presence of invading pathogens influences the subsequent activation and recruitment of inflammatory cells and soluble mediators [27]. Despite the clinical importance of coagulase-negative staphylococci, particularly *S*. *epidermidis*, in PD peritonitis, few studies have directly assessed how mesothelial cells respond to these pathogens. Using an unbiased whole transcriptome approach, coupled to a targeted gene panel with subsequent qPCR validation, we have demonstrated the complexity of the early mesothelial cell response to *S*. *epidermidis* infection, the biological variability inherent in different infecting strains of bacteria, and the limitations of the Met-5A cell line for the study of peritoneal biology.

Our study purposefully focussed on an early period post-infection of 1 hour, due to viability studies demonstrating increased mesothelial cell death after this time with some bacterial species. Even by 1 hour, two signalling pathways related to apoptosis were represented in the top 35 canonical pathways, suggesting that severe infection can activate pathways leading to cell death early after infection.

Analysis of our microarray data revealed 38 genes that were significantly differentially regulated by *S*. *epidermidis* using a stringent cut-off and accounting for multiple comparisons. Of these, 25 genes were up-regulated, 13 genes were down-regulated, and 4 genes were common to both isolates (Up: ZFP36, NFKBIA and MAP3K5; Down: ITLN1). To provide a further, more targeted analysis of gene regulation following infection, we next utilized the Qiagen RT^2^ PCR array to focus on genes known to be associated with antibacterial responses, and to examine their expression after infection with *S*. *epidermidis*. Two profiles of gene expression were observed in primary mesothelial cells exposed to *S*. *epidermidis* reference isolates, with ATCC^®^ 14990 resulting in predominantly down-regulation of genes, and ATCC^®^ 12228 showing more frequent up-regulation of gene expression (Fig 6). The observed variation in mesothelial cell responses to individual isolates of *S*. *epidermidis* may be explained by the high genetic variability present in the genomes of *S*. *epidermidis* isolates [28]. *S*. *epidermidis* generally lack the more common “classical” virulence factors such as toxins [29], and differences in gene content between individual strains have been linked to their ability to invade tissue and cause disease [28]. Three of the differentially regulated genes identified by microarray were present on the RT^2^ PCR array (NFKBIA, TNF, CXCL2), with all 3 genes significantly up-regulated by *S*. *epidermidis* in primary mesothelial cells. Differences were also observed between in responses between primary mesothelial cells and the Met-5A cell line. Given the heterogeneity in results, we conducted a further series of dose-response experiments in both mesothelial cell types using qPCR. qPCR validation was conducted on six genes, including CCL5 (RANTES), which was consistently up-regulated in response to *S*. *epidermidis* infection, and TLR4 and ITLN1, which were down-regulated by primary mesothelial cells in response to multiple *S*. *epidermidis* isolates.

As the immortalised mesothelial cell line Met-5A is commonly employed for the study of mesothelial cell responses, we assessed whether results were comparable between Met-5A and primary mesothelial cells across a number of genes. Primary mesothelial cells exhibited a consistent pattern of TLR4 expression in response to *S*. *epidermidis* infection. The presence of TLR4 mRNA in primary mesothelial cells is consistent with previous studies [30]. Expression of TLR4 is a confirmatory marker for Met-5A cells [31], however discordant TLR4 expression was seen in Met-5A cells. Conflicting data were also seen for TNF expression in Met-5A cells. qPCR data showed uniform down-regulation of TNF in Met-5A cells whereas primary mesothelial cells displayed a strong dose-dependent signal early after infection. Given that Met-5A cells have aberrant TNF and TLR4 expression, caution should be exercised before these cells are used for immunological studies.

Several genes identified through our experimental approach have been linked to roles in host responses to bacterial infection (CCL5, ITLN1), immune modulation (ZFP36, NFKBIA) and damage (EDN1) during PD or during episodes of PD peritonitis [32–34]. CCL5 is a chemokine that is secreted by mesothelial cells and is well-known for its role the recruitment of mononuclear cells during infection [35]. ITLN1, also known as human intelectin-1 or omentin, was uniformly down-regulated by mesothelial cells in response to *S*. *epidermidis* infection. Intelectin has been proposed as a means of microbial surveillance by host cells [32, 33] and the ability of *S*. *epidermidis* to down-regulate intelectin may be a bacterial mechanism of avoiding detection by the host immune system. Intelectin has also been identified by proteomic analyses of PD fluid [34]. ZFP36, encoding tristetraprolin, is a key regulator of cytokine and chemokine expression during inflammation, particularly of TNF [36]. NFKBIA, encoding IκBα, was identified by both microarray and RT^2^ PCR array studies, and forms a negative-feedback loop limiting the magnitude and duration of the inflammatory response [37]. TLR signaling induces a rapid increase in TNF mRNA, and tristetraprolin plays a critical role in eliminating TNF mRNA [38] and preventing an excessive immune response. Endothelin-1, encoded by EDN1, is a vasoconstrictor peptide recently shown to play a role in the induction of fibrosis during PD [39, 40]. Although fibrosis is generally considered a late event in PD [41], peritonitis has been shown to be a risk factor [42, 43], and our results suggest pathways involved in fibrosis are activated early after infection. Up-regulation of endothelin-1 following *S*. *epidermidis* infection may contribute to mesothelial cell dysfunction and the mesothelial-to-mesenchymal transition [39].

Analysis of the most highly regulated mesothelial cell genes following *S*. *epidermidis* infection identified 35 canonical pathways, including TNF, TLR and IL-17A signalling. TNF is potent pro-inflammatory cytokine and mediator of the acute inflammatory response [37]. TNF expression is activated early after infection and signals through the TNFR1 and TNFR2 receptors [44]. TLRs recognise pathogen-associated molecular patterns (PAMPs) on invading microbes, activating downstream pathways and cytokines that are critical to the innate immune response [45, 46]. High levels of IL-17, a potent pro-inflammatory mediator involved in host defence and inflammation [47], have been associated with a protective immune response early in PD peritonitis, correlating with favourable outcomes [48]. IL-17A has been shown to play a key role in PD-induced peritoneal damage, with significantly elevated levels of IL-17A protein detected in effluent from patients on PD for more than 3 years [49]. Furthermore, immunostaining of biopsy specimens has revealed that IL-17A expression, although rarely seen in healthy peritoneal tissue, positively correlated with length of time on PD [49]. Ten of the differentially regulated genes identified by microarray are downstream of TNF, confirming the relevance of this pathway in mesothelial cell responses to *S*. *epidermidis* infection.

There are several limitations to our study that need to be considered. Only a single time-point was assessed, and as changes in gene expression are likely to be highly dynamic, particularly early after infection, this may account for some of the variability seen between isolates. Despite the expectation that infection with a single species of bacteria would provide a clear dominant response in mesothelial cells, marked biological variability was seen with different isolates of bacteria. Comparative genomic studies have revealed the *S*. *epidermidis* genome consists of 80% core genes, and a 20% variable gene pool, which can be exchanged between bacterial species [50, 51]. Genomic variation and the presence of specific virulence factors are likely to contribute to the varying responses of mesothelial cells to different isolates of *S*. *epidermidis*, which may be relevant to clinical outcomes. Future studies examining protein-level changes induced by expression of differentially regulated genes will be important [52]. Additionally, a relatively high inoculum dose of *S*. *epidermidis* was used to mimic a severe peritoneal infection, and growth characteristics and virulence factor expression may be influenced by bacterial density [53, 54].

Compared to previous research in this area, our study has several advantages. Analyses were conducted using both primary mesothelial cells and the widely-employed Met-5A cell line, with results highlighting the need to validate gene expression findings in primary cells. In addition, live clinical isolates of *S*. *epidermidis* cultured from patients with PD peritonitis were used, unlike many studies that have relied on either cell-free extracts [30] or heat-killed microorganisms [50, 55], which may fail to capture the potential complexity resultant from microbial physiological activity.

## Conclusions

Peritonitis remains a major clinical problem for patients on peritoneal dialysis. We have identified a large number of genes and pathways regulated by *S*. *epidermidis* infection, including TNF, TLR4, CCL5, EDN1, ITLN1 and ZFP36. We have highlighted the strain-specific heterogeneity in responses and limitations of Met-5A mesothelial cells, as well as providing insight into the processes shaping the host immune response early after infection. Analysis of how these responses vary over time and between other bacteria causing peritonitis is highly likely to provide an explanation for differences in clinical outcomes and to identify novel therapeutic targets for the treatment of PD peritonitis.

## Supporting information

S1 Table Top canonical pathways. Twenty-eight differentially expressed genes identified by microarray analysis of primary mesothelial cells exposed to *S*. *epidermidis* for 1 hour were analysed by IPA and 35 canonical pathways were represented in our dataset.
(PDF)
