# Supplementary material for: Analysis of early mesothelial cell responses to Staphylococcus epidermidis isolated from patients with peritoneal dialysis-associated peritonitis
Source: PLoS One. 2017 May 24;12(5):e0178151. doi: 10.1371/journal.pone.0178151 (PMC5443531; doi:10.1371/journal.pone.0178151)
Supplement: S1 Table — Twenty-eight differentially expressed genes identified by microarray analysis of primary mesothelial cells exposed to S. epidermidis for 1 hour were analysed by IPA and 35 canonical pathways were represented in our dataset. (PDF) [file pone.0178151.s001.pdf]

S1 Table: Top canonical pathways. Twenty-eight differentially expressed genes identified by microarray analysis of primary mesothelial cells exposed to *S. epidermidis* for 1 hour were analysed by IPA and 35 canonical pathways were represented in our dataset.

IPA Canonical Pathways	B-H adj. *p*-value[1]	Ratio[2]	Molecules
TNFR2 Signaling	1.20E-03	0.11	FOS, NFKBIA, TNF
IL-17A Signaling in Fibroblasts	1.20E-03	0.09	FOS, NFKBIA, NFKBIZ
TNFR1 Signaling	1.86E-03	0.06	FOS, NFKBIA, TNF
CD27 Signaling in Lymphocytes	1.86E-03	0.06	FOS, NFKBIA, MAP3K5
Induction of Apoptosis by HIV1	2.00E-03	0.05	NFKBIA, MAP3K5, TNF
Acute Phase Response Signaling[3]	2.00E-03	0.02	FOS, NFKBIA, MAP3K5, TNF
Production of Nitric Oxide and Reactive Oxygen Species in Macrophages[3]	2.19E-03	0.02	FOS, NFKBIA, MAP3K5, TNF
IL-10 Signaling	2.19E-03	0.04	FOS, NFKBIA, TNF
LPS-stimulated MAPK Signaling	2.19E-03	0.04	FOS, NFKBIA, MAP3K5
Toll-like Receptor Signaling	2.19E-03	0.04	FOS, NFKBIA, TNF
Role of Osteoblasts, Osteoclasts and Chondrocytes in Rheumatoid Arthritis	2.75E-03	0.02	FOS, NFKBIA, MAP3K5, TNF
RANK Signaling in Osteoclasts	2.82E-03	0.03	FOS, NFKBIA, MAP3K5
Apoptosis Signaling	2.82E-03	0.03	NFKBIA, MAP3K5, TNF
PPAR Signaling	2.82E-03	0.03	FOS, NFKBIA, TNF
Death Receptor Signaling	2.82E-03	0.03	NFKBIA, MAP3K5, TNF
Type I Diabetes Mellitus Signaling	4.17E-03	0.03	NFKBIA, MAP3K5, TNF
PKCθ Signaling in T Lymphocytes	4.27E-03	0.03	FOS, NFKBIA, MAP3K5
Type II Diabetes Mellitus Signaling	4.27E-03	0.03	NFKBIA, MAP3K5, TNF
14-3-3-mediated Signaling	4.27E-03	0.03	FOS, MAP3K5, TNF
IL-6 Signaling	4.27E-03	0.03	FOS, NFKBIA, TNF
IL-17A Signaling in Gastric Cells	4.27E-03	0.08	FOS, TNF
Role of Macrophages, Fibroblasts and Endothelial Cells in Rheumatoid Arthritis	4.27E-03	0.01	FOS, NFKBIA, CXCL12, TNF
GNRH Signaling	4.90E-03	0.02	FOS, EGR1, MAP3K5
Cdc42 Signaling	4.90E-03	0.02	FOS, BAIAP2, MYLK
4-1BB Signaling in T Lymphocytes	5.62E-03	0.06	NFKBIA, MAP3K5
CXCR4 Signaling	7.08E-03	0.02	FOS, EGR1, CXCL12
April Mediated Signaling	7.76E-03	0.05	FOS, NFKBIA
Role of PKR in Interferon Induction and Antiviral Response	7.76E-03	0.05	NFKBIA, TNF
B Cell Activating Factor Signaling	7.76E-03	0.05	FOS, NFKBIA
Granulocyte Adhesion and Diapedesis	7.76E-03	0.02	CXCL12, CXCL2, TNF
Tight Junction Signaling	7.76E-03	0.02	FOS, MYLK, TNF
MIF Regulation of Innate Immunity	7.76E-03	0.05	FOS, NFKBIA
B Cell Receptor Signaling	7.94E-03	0.02	NFKBIA, EGR1, MAP3K5
iNOS Signaling	7.94E-03	0.05	FOS, NFKBIA
Agranulocyte Adhesion and Diapedesis	8.13E-03	0.02	CXCL12, CXCL2, TNF

[1] Benjamini-Hochberg adjusted *p*-value < 0.01 is considered statistically significant.
[2] Ratio is the number of significantly differentially genes in our dataset relative to the number of genes in an individual pathway.
[3] Pathways with a z-score of >2.
